# Melanotic neuroectodermal tumor of infancy: Presentation of a case affecting the maxilla

**DOI:** 10.4103/0973-029X.64309

**Published:** 2010

**Authors:** Pooja Agarwal, Susmita Saxena, Sanjeev Kumar, Richi Gupta

**Affiliations:** *Department of Oral Pathology and Microbiology, Subharti Dental College, Meerut, U.P, India*; 1 *Department of Oral and Maxillofacial Surgery, Subharti Dental College, Meerut, U.P, India*

**Keywords:** Benign neoplasm, maxilla, melanotic neuroectodermal tumor of infancy

## Abstract

Melanotic neuroectodermal tumor of infancy is a rare, distinctive neoplasm of early infancy with rapid expansile growth and a high rate of recurrence. Most commonly, the lesion affects the maxilla of infants during the first year of life. One such case was diagnosed in the Department of Oral Pathology and Microbiology in Subharti Dental College, Meerut.

## INTRODUCTION

Melanotic neuroectodermal tumor of infancy (MNTI) is a rare and benign neoplasm of early infancy with rapid expansile growth and a high recurrence rate. In the medical literature, the tumor has been referred to with a variety of synonyms, such as congenital melanocarcinoma,[1] retinal anlage tumor,[2] pigmented congenital epulis[3] or melanotic progonoma.[4] This lesion is found mainly in children below 1 year of age.[5] A small number of cases have been reported in older children and in adults. No predilection in gender was described. The majority (90%) arises in the head and neck and, generally, in the anterior region of the maxilla, but it can also occur at other locations, including the skull, mandible, brain and epididymis.[6]

The lesion is generally accepted to be neural crest in origin.[5]

Clinically, MNTIs are soft and rapidly growing pigmented swellings. They often destroy the underlying bone and may be associated with displacement of developing teeth. Clinical and radiological findings may suggest a diagnosis of MNTI, but histopathological examination is required for a definite diagnosis. MNTI generally follows a benign course, but the rate of recurrence is reported to be 15% within 1 year of initial excision.[7] In this report, we present a case of MNTI of the maxilla and discuss the pathology, review of the literature and clinical course of the disease.

## CASE REPORT

A 6-month-old female patient was referred to plastic and oral and maxillofacial surgeons with a complaint of an expansile growth of the maxilla. The swelling was first noticed 2 months ago and slowly increased to the present size.

**Figure 1:**
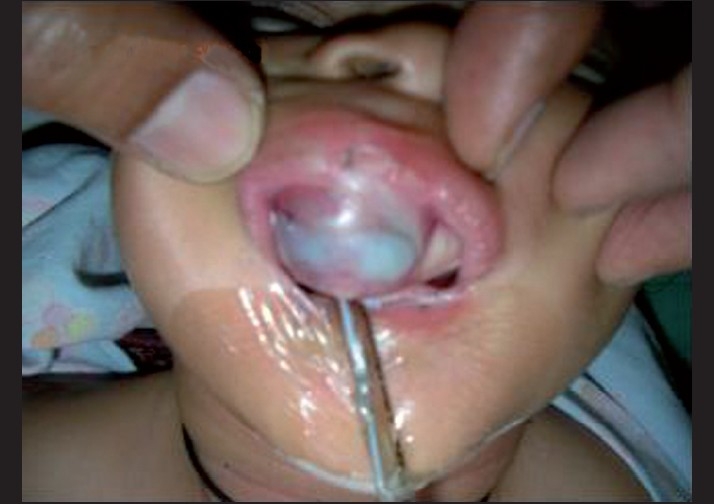
Firm, nonulcerated reddish-blue tumor in the maxilla

Clinical examination showed a normally developed female infant. On the anterior region of the maxilla, a firm nonulcerated reddish-blue tumor of about 4 cm × 3 cm was seen to be protruding through the lips and covered by the intact mucosa [Figure 1].

**Figure 2:**
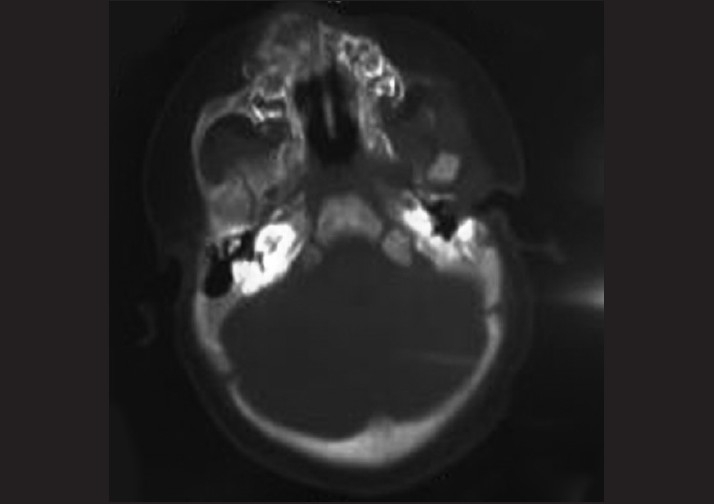
Computed tomography scan showing lobed extensive tumor of the anterior maxilla and central hypodense area

The computed tomography (CT) scans of the head and neck region showed a tumor in the anterior maxilla region with a central hypodense area [Figure 2]. Results of routine laboratory investigations were within normal limits. The urine levels of vanillyl mandelic acid were elevated (6–9.5 mg).

Excisional biopsy of the tumor was undertaken.

**Figure 3:**
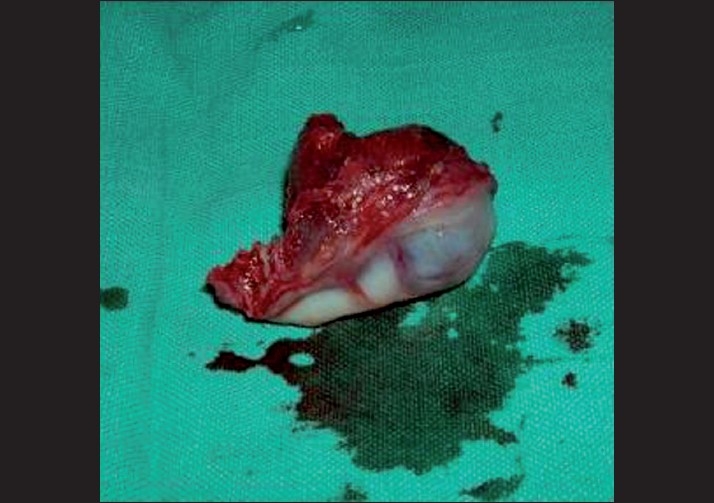
Gross specimen showing a well-circumscribed soft mass

The gross specimen received in the Department of Oral Pathology and Microbiology of Subharti Dental College comprised of a firm, well-circumscribed mass, measuring 4.5 cm × 3 cm × 2.5 cm. The cut surface was grayish-white in color with black pigmented areas [Figure 3].

**Figure 4:**
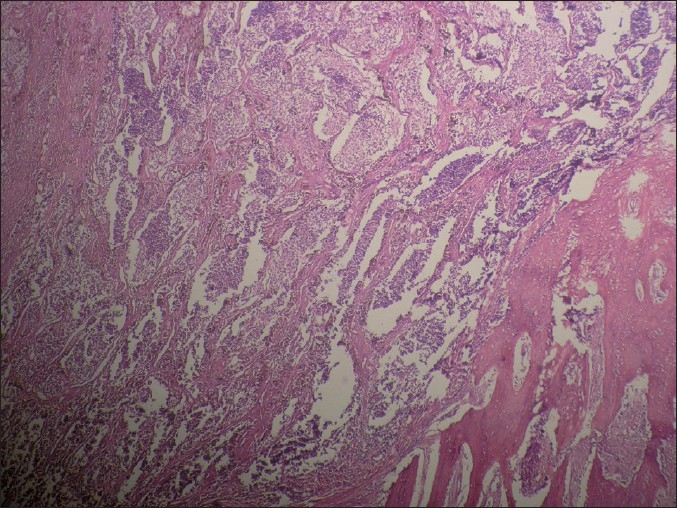
Fibrovascular stroma with nests and clusters of cells arranged in an alveolar pattern (H and E, ×4)

**Figure 5:**
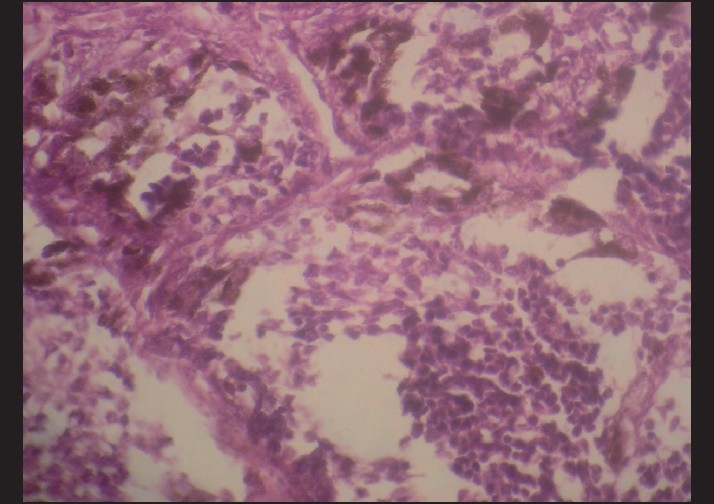
Central part of the alveoli made up of small and round cells with little cytoplasm and dark nuclei whereas the peripheral part comprised of cuboidal, flattened epitheloid cells (H and E, ×4)

**Figure 6:**
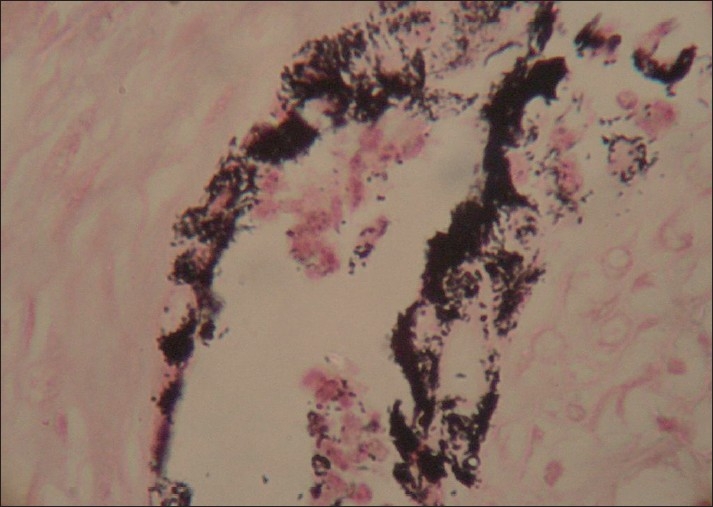
Peripheral melanin-containing epitheloid cells showing positivity (Masson’ Fontana, ×4)

**Figure 7:**
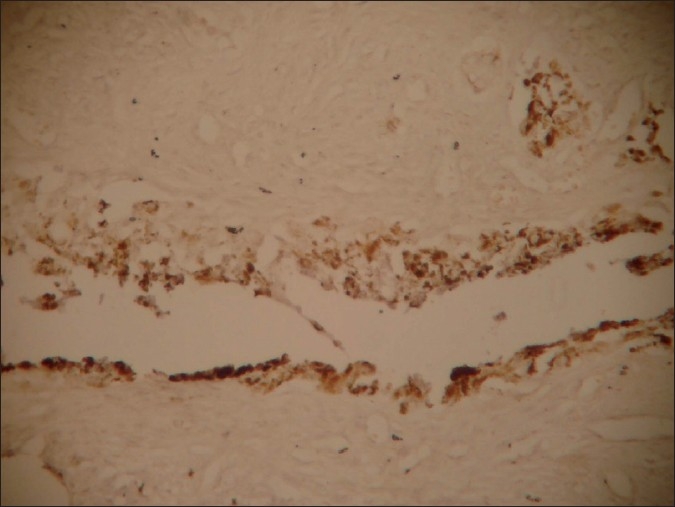
IHC staining showing HMB-45 positivity in peripheral epitheloid cells

**Figure 8:**
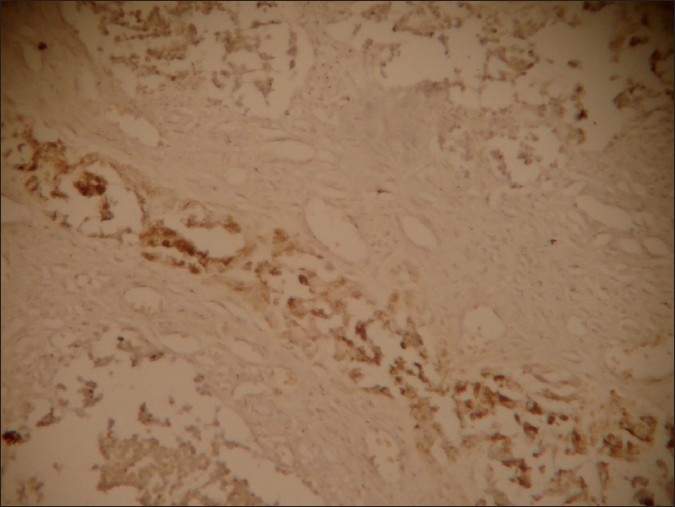
IHC staining showing synaptophysin positivity in central small neuroblast-like cells (×10)

Microscopically, the tumor comprised of nests and clusters of tumor cells arranged in an alveolar pattern separated by fibrovascular stroma. The central part of the alveoli were made up of small and round cells with little cytoplasm and dark nuclei whereas the peripheral part comprised of cuboidal, flattened epitheloid cells containing melanin pigment in the cytoplasm [Figures 4 and 5]. Masson Fontana staining showed positivity for melanin in peripheral epitheloid cells [Figure 6]. With the above features, a diagnosis of primitive neuroectodermal tumor of infancy was made. IHC was advised for confirmation of the diagnosis. Immunohistochemical analysis revealed positive staining for HMB-45 in the melanin-containing peripheral polygonal cells and for synaptophysin in the small neuroblast-like cells [Figures 7 and 8]. Thus, a final diagnosis of MNTI was given.

The patient’s recovery was uneventful and healing progressed satisfactorily.

## DISCUSSION

The first case MNTI to be reported in the literature was designated as “Congenital melanocarcinoma” by Krompecher[1] in 1918. He described a pigmented tumor of the maxilla associated with a developing tooth and elements of dental lamina in a 2-month-old infant. In 1926, Mummery and Pitts[8] reported a case of pigmented maxillary tumor in a 5-month-old girl. The characteristics of the tumor suggested that it arose from some aberration of the dental epithelium, and the term melanotic epithelial odontoma was introduced. Halpert and Patzer[9] reported a similar tumor in 1947, which contained pigmented epithelium and was suggestive to be the ciliary body of the eye. Small unpigmented cells that resemble neuroblasts from the retinal neuroepithelium were also present. They suggested that the tumor arose from the entrapment of the retinal anlage in the embryologic fusion lines of the developing maxilla. Stowens[10] reported three cases of a tumor, which he believed resembled the vomeronasal organ of Jacobson in several ways. In 1966, Borello and Gorlin[5] reported a case of melanotic tumor in the maxilla of a 3-month-old boy. Before surgical removal of the tumor, there was increased urinary excretion of 3-methoxy-4-hydroxymandelicacid (VMA), which returned to normal after the tumor was removed. Because a high urinary level of VMA is also common in other tumors of neural crest origin, Borello and Gorlin believed that this was highly suggestive of a neural crest origin. Also, because this tumor is common in infancy, they recommended the term MNTI.

A high level of urinary VMA is useful for diagnosing tumors of neural crest origin.[5,6]

The levels of VMA return to normal after excision of MNTI,[6,11] as in the present case. However, VMA levels seem to have no relation to the tumor’s biological behavior.[12]

The MNTI clinically presents as a rapidly growing, painless, expansile, unencapsulated partly pigmented mass, typically in the maxillary region.[13] It tends to occur as a single lesion. However, multiple lesions have also been reported.[9]

Conventional radiographs of bony lesions usually show radiolucency with or without irregular margins. It is typical of CT scans to reveal hyperdense masses, but hypodense variants have been reported as well. The CT can accurately define the extent of the lesion and thus provides a good basis for surgical planning. Magnetic resonance imaging shows a hypodense mass with focal areas of hyperdensity.[7]

In addition to the typical clinical presentation, the cytology and histology are distinctive, showing a dual population of small neuroblastic cells and larger melanin-containing epithelial cells,[14] as also seen in our case.

IHC markers are helpful in differentiating MNTI from embryonal rhabdomyosarcoma (desmin and myoglobin positive), Burkitt’s lymphoma (common leukocyte antigen positive) and malignant melanoma (HMB-45 and S-100 positive).[15,16]

Immunohistochemically, reactivity for HMB-45 and synaptophysin in our case indicate melanocytic and neuroblastic differentiation of the tumor cells, confirming the diagnosis of MNTI.

The treatment of choice consists of complete surgical excision. Individuals with MNTI that are not amenable to surgical management alone may receive other modes of treatment.[9] In general, this may be chemotherapy alone, chemotherapy with radiotherapy, chemotherapy before and after the surgical treatment, radiotherapy and surgical treatment or a combination of all. Chemotherapy may serve as an alternative or adjuvant option in the treatment of widely extended MNTIs.[7,17]

## CONCLUSION

Although MNTI behaves in a benign fashion, recurrences can occur especially within the first 6 months with the need for close follow-up postoperatively. Early detection and treatment will avoid further complications and may support a favorable outcome for the patient.

In the present case, early diagnosis and treatment prevented further complications and the patient was followed-up for 5 months without any recurrence.
